# Magnitude and factors associated with anemia among pregnant women attending antenatal care in public health centers in central zone of Tigray region, northern Ethiopia: a cross sectional study

**DOI:** 10.1186/s12884-018-2063-z

**Published:** 2018-11-01

**Authors:** Teklit Grum, Ermyas Brhane, Solomon Hintsa, Gizienesh Kahsay

**Affiliations:** 1 Department of Reproductive Health, College of Health sciences, Aksum University, Aksum, Ethiopia; 2 Department of Human Nutrition, College of Health sciences, Aksum University, Aksum, Ethiopia; 3 Department of Epidemiology and Biostatistics, College of Health sciences, Aksum University, Aksum, Ethiopia

**Keywords:** Anemia, Pregnant women, Ethiopia

## Abstract

**Introduction:**

Anemia is defined as a low blood hemoglobin concentration (< 11 mg/dl). It is a global public health problem especially in pregnant women and is associated with higher risk for both maternal and perinatal mortality and morbidity. In developing countries, like Ethiopia where anemia is common, determining the magnitude and identifying factors that are associated with anemia is necessary to control it.

**Methods:**

Facility based cross sectional study design were conducted among 638 pregnant women attending antenatal care in public health centers in central zone of Tigray region, Northern Ethiopia from November 1/2017 to January 30/2018 using stratified multi stage sampling method. The data was collected through interviewing the pregnant women face to face after getting informed consent using structured and pre-tested questionnaire. The data was coded and entered in to Epi-info 7 then exported to Stata 14 for cleaning and further analysis. Both Bivariable and multi variable logistic regression model was used in the data analysis.

**Results:**

The overall magnitude of anemia (hemoglobin level < 11 mg/dl) were found that 16.88% (95% CI: 13.95%, 19.8%). Factors which were significantly associated with anemia in the multivariable analysis were: history of malaria attack 1 year prior to study period (AOR = 4.73, 95% CI: 2.64, 8.46), women who had history of excessive menstrual bleeding (AOR = 3.94, 95% CI: 2.11, 7.35), unplanned pregnancy (AOR = 2.5, 95% CI: 1.4, 4.42) and three times or less meal frequency (AOR = 1.89, 95% CI: 1.02, 3.5).

**Conclusion:**

The magnitude of anemia among pregnant were found that 16.88%. Malaria attack, excessive menstrual bleeding, pregnancy planning and meal frequency were found that significantly associated with anemia in the multivariable analysis. Pregnant women are recommended to increase meal frequency. Health providers should give attention to pregnant women who had history of malaria attack, excessive menstrual bleeding and women whose pregnancy were not planned.

## Introduction

Anemia is defined as a low blood hemoglobin concentration [1]. It is a global public health problem affecting people in all age groups with major consequences for human health as well as social and economic development [1–3]. Anemia is a common health problem in pregnant women which is wider in developing countries than developed countries. As a result, it is associated with higher risk of low birth weight and both maternal and perinatal mortality and morbidity [4–7].

In developing countries, pregnant women are prone to anemia due to low socioeconomic conditions. The poor nutritional intake, repeated infections, frequent pregnancies and low health-seeking behaviors are associated with anemia [5, 8].

Since anemia is more common in child bearing women especially during pregnancy in Ethiopia, national nutritional strategies are formulated to reduce as part of the health sector transformation plan [9]. In countries like Ethiopia where anemia due to nutritional deficiencies mainly related to iron deficiency are the major causes [10, 11], identifying factors that are associated with anemia is necessary to control it. So, this study is aimed at assessing the magnitude and factors associated with anemia in pregnant women attending antenatal care.

## Methods

### Study design

Facility based cross sectional study design was conducted.

### Study area and period

The study was conducted in central zone of Tigray region, Northern Ethiopia from November 1/2017 to January 30/2018. Central zone of Tigray is one of the six zones found in Tigray region. According to the 2007 census, Central zone of Tigray has 12 districts with estimated population of 1,500,000. Out of these population 750,000 are females and 750,000 are males. Central zone of Tigray has 66 health facilities (one referral hospital, 3 general hospitals, 6 primary hospitals and 56 health centers). The antenatal care (ANC) coverage of zone administration is 90%.

### Source population

All pregnant women who attend ANC in public health centers in central zone of Tigray region during the study period.

### Study population

All pregnant women who attend ANC at randomly selected public health centers in central zone of Tigray region during the study period.

### Inclusion and exclusion criteria

#### Inclusion criteria

Pregnant women who attend 1st ANC visit and above at the same health center. Hence hemoglobin level of pregnant women was taken from base line assessment during 1st ANC visit as routine activities before starting any intervention.

#### Exclusion criteria

Women who attend 1st ANC other than the selected health center.

### Sample size determination

The sample size was calculated using single proportion formula using epinfo-7 from study conducted in north west Ethiopia where the proportion of women with anemia was 25.2% [12].

Therefore, the total sample size was calculated using the assumption of marginal error 0.05, and 95% confidence interval. Based on these assumptions, the sample size was estimated as 290. After multiplying by 2 for design effect and adding 10% of non-respondents, the final sample size was determined as 638 study subjects.

### Sampling procedures

Stratified multi stage sampling method was used to select health centers found in the central zone of Tigray region. During the selection of health centers, we stratified them as rural and urban. Two health centers each were randomly selected from 3 randomly selected woreda and one health center from urban in the central zone were included in the study. Proportion to sample size allocation was used to allocate the number of pregnant women from each health facilities by taking 3 months case load pregnant women on ANC visit. Systematic random sampling technique was used to get the study units.

### Data collection procedure and tools

The data was collected through interviewing the pregnant women face to face after getting informed consent using structured and pre-tested questionnaire. The questionnaire was originally developed in English and then translate into Tigrigna (local language). Later on it was translated back to English to ensure its consistency. Finally, it was prepared in Local language (Tigrigna language) to collect data. In addition to that chart review was conducted to extract hemoglobin level at ANC 1st visit and another necessary data from ANC longitudinal register. During data collection, two BSC Degree and 7 diploma holder in nursing were hired as supervisors and data collectors respectively.

### Data analysis

The data was coded and entered in to Epi-info 7 then exported to Stata 14 for cleaning and further analysis. Both Bivariable and multi variable logistic regression model was used in the data analysis. The assumption of logistic regression model fitness was checked using Hosmer and Lemeshow goodness of fit test statistics. Variables with *P*-value < 0.05 in the Bivariable logistic regression analysis was considered for inclusion in the multivariable logistic regression analysis to control the effect of confounders.

Variables which were significantly associated with the outcome variable were declared when adjusted odds ratio (AOR) with 95% confidence interval was significant in the multivariable analysis at *P*-value< 0.05.

### Data quality

Three days intensive training were given to data collectors and supervisors on the data collection tools and collection procedures by the principal investigator.

Daily supervision of data collectors were made at each health centers during the study period by the supervisors and principal investigator. The collected data were carefully checked for completeness and consistency. Any confusion on the data collection procedure and responses were handled timely.

## Results

### Socio-demographic and economic characteristics of study participants

A total of 634 pregnant women were interviewed with the response rate of 99%. The average mean age of study participants was 26.99 years with standard deviation (SD) of 5.99. The majority of age group belongs to 20–34 years which accounts 487(76.81%). Nearly all 609(96.06%) of study participants were with orthodox religion. Regarding to marital status, 13(2.05%) of study participants were not married whereas 552(87.07%) were married. Eighty five (13.41%) of participants were with housewife occupation and 277(43.69%) of the study participants were with primary educational level. Four hundred fifteen (65.46%) of the study participant’s household size were 1–4 and about a quarter of study participants 127(25.08%) were with second wealth quintile (Table 1).

**Table 1:** Socio-demographic and economic characteristics of pregnant women attending ANC in public health centers in central zone of Tigray region, northern Ethiopia, 2018

Variables	Frequency	Percentage
Age in years		
15–20	55	8.68
20–34	487	76.81
≥35	92	14.51
Mean = 26.99 (SD = 5.99)		
Religion		
Orthodox	609	96.06
Muslim	25	3.94
Marital status		
Not married	13	2.05
Married	552	87.07
Divorced	20	3.15
Separated	46	7.26
Others	3	0.47
Occupation		
House wife	85	13.41
Farmer	399	62.93
Merchant	89	14.04
Employee	43	6.78
Others	18	2.84
Level of education		
No formal education	173	27.29
Primary	277	43.69
Secondary	126	19.87
Diploma and above	58	9.15
Household size		
1–4	415	65.46
5–7	181	28.55
≥8	38	5.99
Wealth quintile		
Lowest	127	20.03
Second	159	25.08
Middle	181	28.55
Fourth	60	9.46
Highest	107	16.88

Out of the total study participants, 175(27.6%) were nulliparous and 79(16.92%) had history of abortion. Fifty seven (12.42%) and 37(8.06%) of the study participants were with less than 2 years birth interval and had history of still birth respectively. Regarding to history of malaria attack 1 year prior to this study period, 112(17.67%) of the study subjects had history of malaria. Most of the participants 542(85.49%) and 582(91.8%) had no history of excessive menstrual bleeding and pregnancy related complications respectively. Only 132(20.82%) of study subjects were with planned pregnancy. Almost half 315(49.68%) of the participants were with MUAC less than 23 cm.

The magnitude of anemia (hemoglobin level < 11 mg/dl) were found that 16.88% (95% CI: 13.95%, 19.8%). The mean hemoglobin level of study participants were 12.3 (SD ± 1.33). Most of the pregnant women 489 (77.13%) who attend their first ANC were at the 3–6 months of gestational age (Table 2).

**Table 2:** Obstetric history characteristics of pregnant women attending ANC in public health centers in central zone of Tigray region, northern Ethiopia, 2018

Variables	Frequency	Percentage
Parity		
Nulliparous	175	27.60
1–4	391	61.67
≥5	68	10.73
Ever had abortion		
No	388	83.08
Yes	79	16.92
Birth interval		
< 2 years	57	12.42
≥2 years	402	87.58
Had ever still birth		
No	422	91.94
Yes	37	8.06
Have got malaria in previous year		
No	522	82.33
Yes	112	17.67
Had excessive menstrual bleeding		
No	542	85.49
Yes	92	14.51
Had pregnancy related complication		
No	582	91.80
Yes	52	8.20
Planned pregnancy		
No	132	20.82
Yes	502	79.18
Nutritional status (MUAC)		
< 23 cm	315	49.68
≥23 cm	319	50.32
Hemoglobin level		
< 11 mg/dl	107	16.88
≥11 mg/dl	527	83.12
Mean 12.38(SD ± 1.33)		
HGB level by gestational age (Average HGB level)		
< 3 Months Mean = 12.2(SD ± 1.63)	19	3
3–6 Months Mean = 12.22(SD ± 1.29)	489	77.13
> 6 Months Mean = 12.13(SD ± 1.41)	126	19.87

The meal frequency of study participants with greater than three times per day were 252(39.75%). Majority 541(85.33%) of the total participants were reported that they consume food made from cereals and grains daily. Besides, 179(28.23%) of the total participants didn’t take tea or coffee. Concerning to fruit and green leafy vegetables intake, only 11(1.74%) and 40(6.31%) of the total study participants reported with daily intake respectively (Table 3).

**Table 3:** Dietary factors characteristics of pregnant women attending ANC in public health centers in central zone of Tigray region, northern Ethiopia, 2018

Variables	Frequency	Percentage
Meal frequency		
≤3times per day	382	60.25
>3times per day	252	39.75
Eating food made from cereals, grains		
Daily	541	85.33
Weekly	84	13.25
Monthly or above	9	1.42
Drinking tea or coffee		
No	179	28.23
Before meal	73	11.51
Within 1 h	249	39.27
After 1 h	133	20.98
Fruit intake		
No	291	45.9
Daily	11	1.74
Weekly	266	41.96
Monthly or above	66	10.41
Green leafy vegetables intake		
No	91	14.35
Daily	40	6.31
Weekly	463	73.03
Monthly or above	40	6.31
Dairy products/ milk product intake		
No	297	46.85
Daily	58	9.15
Weekly	211	33.28
Monthly or above	68	10.73
Meat intake		
No	100	15.77
Daily	16	2.52
Weekly	172	27.13
Monthly or above	346	54.57
Egg intake		
No	132	20.82
Daily	62	9.78
Weekly	343	54.10
Monthly or above	97	15.30

Socio-demographic factors like; age of women, religion, marital status, occupation, household size and wealth quintile were not significantly associated with the anemia in Bivariable analysis at *P*-value < 0.05. Similarly, parity of women, history of abortion, birth interval, history of still birth and nutritional status were not significantly associated with anemia in Bivariable analysis. Regarding to dietary intake, variables which were not significantly associated with anemia in Bivariable analysis at *P*-value < 0.05 were; eating food made from cereals and grains, drinking tea or coffee, fruit intake, green leafy vegetables intake, dairy products/ milk product intake, meat intake and egg intake.

Variables which were significantly associated with anemia in Bivariable analysis but remains insignificant in multivariable analysis were; women’s level of education, birth interval and had pregnancy related complication. However, history of malaria attack 1 year prior to study period, women who had history of excessive menstrual bleeding, planned pregnancy and meal frequency were significantly associated with anemia in the multivariable analysis. Women with history of malaria attack were significantly associated with anemia comparing to women who had no malaria attack (AOR = 4.73, 95% CI: 2.64, 8.46). Comparing to women who had no history of excessive menstrual bleeding, anemia was 3.94 times higher in women with history of excessive menstrual bleeding (AOR = 394, 95% CI: 2.11, 7.35). Similarly anemia was higher in pregnant women who had no pregnancy planning (AOR = 2.5, 95% CI: 1.4, 4.42) comparing to their counter parts. Meal frequency less than or equal to 3 times per day was also significantly associated with anemia (AOR = 1.89, 95% CI: 1.02, 3.5) (Table 4).

**Table 4:** Bivariable and multivariable analysis of factors associated with anemia among pregnant women attending ANC in public health centers of central zone of Tigray region, northern Ethiopia, 2018

Variables	Anemia		COR (95%, CI)	AOR (95%, CI)
	Yes	No		
Women’s level of education				
No formal education	39(36.45%)	134(25.43%)	1	1
Primary	35(32.71%)	242(45.92%)	2.01(1.22, 3.33)^*^	1.53(0.81, 2.89)
Secondary	20(18.69%)	106(20.11%)	1.54(0.89, 2.8)	0.98(0.43, 2.25)
Diploma and above	13(12.15%)	45(8.54%)	1.01(0.49, 2.06)	0.52(0.17, 1.61)
Birth interval				
< 2 years	16(20.78%)	41(10.73%)	2.2(1.2, 4.13) ^*^	0.57(0.27,1.2)
≥2 years	61(79.22%)	341(89.27%)	1	1
Malaria attack in last 1 year				
No	64(59.81%)	458(86.91%)	1	1
Yes	43(40.19%)	69(13.09%)	4.46(2.8, 7.1) ^*^	4.73(2.64, 8.46) ^*^
Had excessive menstrual bleeding				
No	69(64.49%)	473(89.75%)	1	1
Yes	38(35.51%)	54(10.25%)	4.82(2.0, 7.84) ^*^	3.94(2.11,7.35) ^*^
Had pregnancy related complication				
No	93(86.92%)	489(92.79%)	1	1
Yes	14(13.08%)	38(7.21%)	1.94(1.01, 3.72) ^*^	1.87(0.77, 4.54) ^*^
Planned pregnancy				
No	38(35.51%)	94(17.84%)	2.54(1.61, 4.0)	2.5(1.4,4.42) ^*^
Yes	69(64.49%)	433(82.16%)		1
Meal frequency				
≤3 times per day	74(69.16%)	308(58.44%)	1.6(1.02, 2.49)	1.89(1.02, 3.5) ^*^
>3times per day	33(30.84%)	219(41.56%)	1	1

*COR* Crude Odds Ratio, *AOR* Adjusted Odds Ratio

*p-Value < 0.05

## Discussion

We conducted study aimed on determining magnitude of anemia and factors associated with it. Out of 634 pregnant women included in the study, 107 (16.88%) were found to be anemic with 95% CI of 13.95 to 19.8% which is lower that studies conducted in Woldia (39.1%) [13], Gode town (56.8%) [11], Butajira (27.6%) [5], Nekemt (52%) [14], Mizan Tepi (23.5%) [15], Dera (30.5%) [4] and North West Tigray (36.1%) [16], but similar with the study conducted in Mekelle (19.3%) [10]. Overall magnitude of anemia in pregnant women is currently decreasing due to multi-sectorial interventions like increasing health access and economy in the country through time but still it remains a public health problem.

In this finding, pregnant women who had history of malaria attack in the last 1 year prior to study period were found that significantly associated with anemia (AOR = 4.73, 95% CI: 2.64, 8.46). This finding is similar with the study conducted in North Western zone of Tigray [16], in Dera District, South Gondar Zone [4] and Sunyani Municipal Hospital, Ghana [17] among pregnant women which declared that malaria attack was significantly associated with anemia. This could be explained that parasitic infections especially malaria results destruction of red blood cells [2].

In this study pregnant women with history of excessive menstrual bleeding were 3.94 times more likely to be anemic than those who had normal menstrual bleeding. A study conducted in Mizan Tepi University Teaching Hospital, South West Ethiopia [15] were also reported that heavy menstrual bleeding was significantly associated with anemia. This may be due to low iron reserves following excess bleeding during menstruation period.

Planned pregnancy was found that significantly associated with anemia. Anemia was 2.5 times higher in pregnant women whose pregnancy was not planned (AOR = 2.5, 95% CI: 1.4, 4.42). Women with their planned pregnancy may prepare prior or early in pregnancy on nutritional intake.

Meal frequency with 3 times or less per day was associated with anemia (AOR = 1.89, 95% CI: 1.02, 3.5). This study is consistent with Studies conducted in Mekelle town [10] and in North West of Tigray [16]. This implies increased meal frequency during pregnancy needs to fulfill the nutrients demand of pregnant women.

## Conclusion

The magnitude of anemia among pregnant were found that 16.88. Factors that were independently significant with anemia were; history of malaria attack, excessive menstrual bleeding, pregnancy which was not planned and three or less meal frequency per a day. It is recommended that pregnant women should increase meal frequency. Health providers should give attention to pregnant women who had history of malaria attack, excessive menstrual bleeding and women whose pregnancy were not planned to control malaria in pregnancy.

## Abbreviations

ANC: Antenatal care
AOR: Adjusted odds ratio
CI: Confidence interval
CM: Centimeter
COR: Crude odds ratio
HGB: Hemoglobin
MUAC: Middle upper arm circumference
SD: Standard deviation
